# Body Surface Gastric Mapping Improves Diagnosis of Gastric Motility Disorders

**DOI:** 10.1007/s11894-026-01049-y

**Published:** 2026-07-20

**Authors:** Jarongkorn Sirimongkolkasem, Christopher N. Andrews, Gregory O’Grady

**Affiliations:** 1 Division of Gastroenterology & Hepatology, Stanford University, Palo Alto, CA USA; 2 Department of Medicine, King Chulalongkorn Memorial Hospital and Center of Excellence in Neurogastroenterology and Motility, Chulalongkorn University, Bangkok, Thailand; 3 Division of Gastroenterology, University of Calgary, Calgary, Canada; 4 Department of Surgery, University of Auckland, Auckland, New Zealand

**Keywords:** Gastric motility, Gastroparesis, Functional dyspepsia, Chronic nausea and vomiting, Interstitial cells of Cajal, Biomarkers, Clinical utility

## Abstract

**Purpose of Review:**

Chronic gastroduodenal symptoms affect > 7% of adults, yet current diagnostic frameworks are challenged by limited mechanistic specificity, weak symptom correlation, and variable reproducibility. This review evaluates how body surface gastric mapping (BSGM), a non-invasive technology combining high-resolution gastric myoelectrical recording with validated symptom profiling, may improve the diagnosis and management of gastric motility disorders.

**Recent Findings:**

BSGM employs a high-resolution 64-electrode array to derive validated biomarkers of gastric motor function, including rhythm stability, frequency, and amplitude, alongside standardised digital symptom and psychometric profiling. A recent international consensus (‘Auckland Classification v1.0’), derived from over 50 published studies and 4,500 + clinical tests, defined six BSGM phenotypes encompassing putative mechanisms of neuromuscular dysfunction, visceral hypersensitivity, centrally-mediated symptoms, and small bowel contributions. BSGM significantly increases diagnostic yield for motility disorders and appears synergistic with gastric emptying testing in joint studies. Observational data currently indicate that BSGM-guided management changes clinical decisions in ~ 80% of patients and is associated with reduced healthcare utilisation. Key benefits include capability to aid discrimination of motor vs sensory disorders, with specific phenotypes provisionally linked to differential treatment responses. Paediatric studies show concordant phenotype patterns, with BSGM dysrhythmia identified as a more severe phenotype.

**Summary:**

BSGM provides mechanism-based phenotyping that extends gastroduodenal evaluation beyond symptom classifications and gastric emptying status. Prospective validation of phenotype-guided treatment algorithms is now underway, and integrated multimodal diagnostic frameworks incorporating BSGM alongside complementary investigations are likely to reshape the clinical approach to these challenging disorders.

## Introduction

Chronic gastroduodenal symptoms are common, affecting approximately 7–8% of adults worldwide [1]. Patients are generally classified into functional dyspepsia and gastroparesis as distinct syndromes, however, these disorders lie on a disease spectrum [2, 3]. In principle, gastroparesis is more strongly linked to neuromuscular injury, including immune activation, interstitial cell of Cajal (ICC) loss, pyloric pathway dysfunction, and fibrosis, whereas functional dyspepsia more often reflects impaired accommodation, abnormal intra-gastric meal distribution, and visceral hypersensitivity or pain syndromes [2, 4, 5]. However, these distinctions are now recognised as somewhat artificial due to overlapping underlying pathophysiologies [3, 6].

Clinically, gastroparesis is often dominated by nausea and vomiting, whereas functional dyspepsia is more often characterized by epigastric pain or burning, postprandial fullness, and early satiety; however, these symptoms also overlap [7]. Patients with persistent or severe symptoms often undergo repeated testing, dietary restriction, medication cycling, and, in some cases, nutritional support without a clear treatment target [7]. The clinical challenge is not simply assigning a syndrome label but identifying the dominant mechanism and treating it accordingly.

In practice, delayed gastric emptying remains the main test-based feature separating gastroparesis from functional dyspepsia. Gastric emptying scintigraphy (GES) remains the standard test for defining gastroparesis, but interpretation is affected by protocol variability in meal composition, imaging duration, and reference ranges [8–10]. Symptom severity tracks at best weakly with emptying [11], with meal-related symptoms showing essentially no correlation with gastric emptying rate in a large functional dyspepsia/idiopathic gastroparesis cohort (r = 0.06) [12], and gastric retention failing to correlate with GCSI severity in a multicenter tertiary-care cohort [7]. Reproducibility is also limited: despite stable symptoms, 37%−42% of patients were reclassified between functional dyspepsia and gastroparesis on repeat gastric emptying testing over 48 weeks [7], although reproducibility is also impacted by meal size and composition, diagnostic cut-offs and measurement of timepoints [13]. Gastric emptying is clinically important, but it is an integrative outcome rather than a mechanism-specific biomarker.

This diagnostic gap highlights the need for mechanism-based classification. Body surface gastric mapping (BSGM) was developed toward this goal, combining high-resolution (HR) gastric myoelectrical mapping with symptom tracking during a standardized meal and psychometric assessment. By integrating these data, BSGM helps identify physiologic phenotypes beyond gastric emptying status alone [14–16]. This review evaluates how BSGM-based phenotyping may guide clinical management in patients with gastric motility disorders.

### BSGM: Clinical Principles

Gastric motility is coordinated by bioelectrical slow waves generated by ICC, in concert with neurohormonal co-regulation [17]. However, gastric slow waves conduct weakly to the body surface (amplitude ~ 100 × lower than cardiac), such that reliable clinical applications have only recently emerged with modern HR approaches [15]. BSGM should therefore be distinguished from earlier low-resolution techniques (electrogastrography; EGG), which failed to achieve routine clinical adoption owing to poor signal-to-noise ratio and weak specificity relative to high-definition techniques (≳32 sensors) [15, 18].

BSGM is a non-invasive, radiation-free, office-based diagnostic aid currently implemented through one FDA-cleared commercial system (Gastric Alimetry, New Zealand). This system employs a 64-electrode patch (8 × 8 electrodes) to maximise gastric signal capture, together with neural-networks that eliminate noise [19]. The flexible patch is placed on the epigastrium connected to a compact wearable reader, with a standardized protocol spanning 4.5 h (30 min baseline, followed by a 450kCal nutrient drink and bar meal, and 4-h of post-prandial recordings) [15]. During the test, the patient sits in a chair and logs their symptoms at ~ 15-min intervals into a validated app, capturing both continuous symptoms and ‘events’ (e.g. vomiting, belching) [20]. A clinical report is then delivered via a cloud-based platform.

A key principle of BSGM is that it integrates motor, sensory, and psychological assessment within a single session, rather than providing physiological data in isolation. The motor assessment primarily comprises three validated spectral metrics with established normative reference intervals [21, 22]. These are the Principal Gastric Frequency (PGF, reflecting the underlying pacemaker rhythm), Gastric Alimetry Rhythm Index (GA-RI, quantifying rhythm stability independently of frequency), and the BMI-adjusted amplitude (quantifying both baseline power and contractile response). These metrics show reliable short- and long-term test–retest reproducibility [23]. More recently, a ‘Meal Response Ratio’ has also been proposed, characterising the timing of the postprandial gastric response [24].

**Fig. 1:**
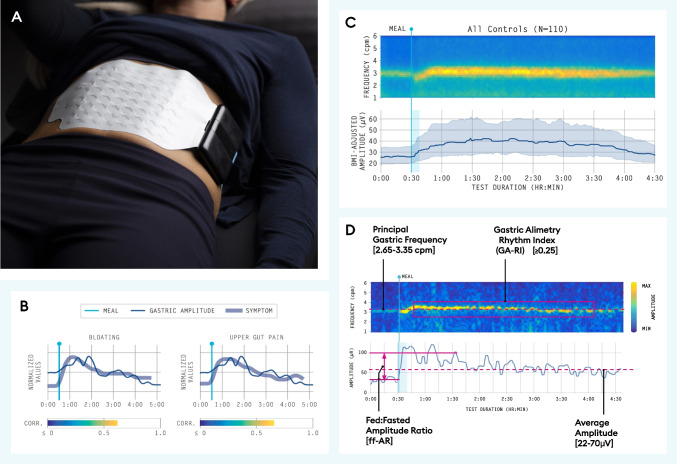
Body surface gastric mapping (BSGM) system and outputs. **A** The Gastric Alimetry system in use, showing the flexible 64-electrode array placed on the epigastrium and connected to a compact wearable reader. **B** Symptom-physiology correlation analysis, illustrating how continuous symptom profiles (bloating, upper gut pain) are mapped against gastric amplitude over the test duration. Correlation coefficients (color bar) quantify concordance or discordance between symptoms and gastric motor activity. **C** Normative spectral data from 110 healthy controls, showing the frequency spectrogram (upper) and BMI-adjusted amplitude trace with confidence intervals (lower) across the 4.5-h test protocol (30-min fasting baseline followed by a standardized meal). **D** Summary of the principal BSGM spectral metrics and their normative reference intervals: Principal Gastric Frequency (PGF; 2.65–3.35 cpm), Gastric Alimetry Rhythm Index (GA-RI; ≥ 0.25), Fed:Fasted Amplitude Ratio (ff-AR), and Average Amplitude (22–70 µV). [Modified with permission from Varghese et al, American Journal of Gastroenterology (21)]

The sensory assessment correlates validated symptom profiles against gastric amplitude and their timing across the test, enabling identification of symptom–physiology concordance or discordance [25]. A validated psychological screening instrument is also optionally administered at the start of the test to screen for gut-brain associations, covering depression, anxiety and stress domains [26]. Figure 1 shows BSGM in operation with example outputs.

### BSGM Phenotypes

By integrating these motor, sensory, and psychological data streams, BSGM enables patient phenotyping that moves beyond syndrome labels toward mechanism-based classification. An international Working Group of over 45 clinicians and researchers recently convened to formalise this approach, producing the “Auckland Classification v1.0”, the first consensus classification scheme for BSGM-defined phenotypes in chronic gastroduodenal disorders [27]. The Classification was derived from a systematic review of 50 + published studies, a multicentre database of over 4,500 + tests, and a modified Delphi consensus process, with 10 of 11 statements achieving > 80% agreement.

**Fig. 2:**
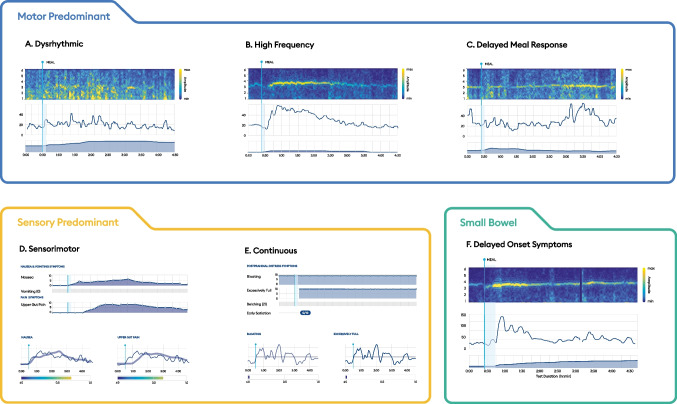
BSGM phenotypes defined by the recent Auckland Classification v1.0. Six phenotypes are shown, grouped by predominant mechanism. **Motor-predominant phenotypes:A** Dysrhythmic—characterized by unstable slow wave activity (low GA-RI) with spectral scatter; **B** High Frequency—showing elevated Principal Gastric Frequency with preserved rhythm stability; **C** Delayed Meal Response—demonstrating weak or delayed postprandial gastric activity. **Sensory-predominant phenotypes: D** Sensorimotor—defined by tight temporal correlation (> 0.5) between symptom curves (nausea, upper gut pain) and gastric amplitude; **E** Continuous—showing persistent high symptom burden (bloating, excessive fullness) independent of meal ingestion or gastric motor activity. **Small-bowel-predominant phenotype: F** Delayed Onset Symptoms—characterized by symptom emergence after the majority of postprandial gastric activity has subsided. Each panel displays the frequency spectrogram (upper) and either symptom–amplitude overlay or amplitude trace (lower) from representative patient recordings. Phenotypes may overlap within individual patients. [Reproduced from Varghese et al under a Creative Commons Attribution 4.0 International License (CC BY 4.0)] (27)

Six phenotypes were resolved, comprising three motor predominant, two sensory predominant, and one small-bowel predominant groups (Fig. 2). These may overlap.

*Motor-predominant phenotypes:*

- Dysrhythmic: Defined by sustained low GA-RI, indicating irregular slow wave activity. This phenotype is linked to ICC depletion and neuromuscular dysfunction, now established as a key hallmark in a substantial patient subgroup with chronic gastric symptoms regardless of gastric emptying status [4, 28]. This phenotype is currently considered to comprise a more severe patient subgroup, and may indicate higher symptom burdens and health needs [29–32].
- High Frequency. Defined by elevated PGF with preserved rhythm stability. In emerging work, this phenotype has been linked to vagal neuropathy or injury, particularly in long-standing diabetes, and may signal a more treatment-refractory cohort [33, 34].
- Low Meal Response. Defined by weak or delayed postprandial gastric activity (Meal Response Ratio < 1) with meal-induced symptoms, and associated with delayed gastric emptying [24]. Confidence around this biomarker and phenotype is still emerging, currently scoring lower on expert consensus [27].

*Sensory-predominant phenotypes:*

- Sensorimotor. Defined by tight correlation between symptom curves and gastric amplitude (correlation > 0.5); and associated with anxiety and hypervigilance [25].
- Continuous. Defined by persistent high symptom burden that is independent of meal ingestion or gastric activity, and consistently associated with psychological comorbidity (depression, stress, anxiety) [25, 35, 36].

*Small-bowel-predominant phenotype:*

- Delayed Onset Symptoms. Defined by symptoms emerging after the majority of postprandial gastric activity has subsided, suggesting a distal contribution to symptom genesis [25, 37].

It should also be noted that the first BSGM classification scheme includes additional emerging phenotypes, and the scheme is expected to evolve as further evidence emerges from this new diagnostic modality.

### Clinical Impact of BSGM in Diagnosis and Management

The principal clinical value of BSGM lies in its ability to detect abnormalities that GES may miss. Among patients who underwent both tests, BSGM detected significantly more abnormalities than GES alone (33.3% vs 22.7%), including abnormal gastric electrical activity in some patients without delayed emptying. Combined testing increased the diagnostic yield to 42.7%, suggesting the two modalities are complementary [38].

BSGM also adds value through symptom phenotyping, with the diagnostic yield rising to 62.7% when all phenotypes were considered in the above study [38]. Symptoms that correlate with gastric activity suggest a sensorimotor pattern and may support accommodation- or hypersensitivity-directed therapy, whereas continuous symptoms that occur independently of gastric activity may suggest a gut-brain mechanism and may favor neuromodulation or psychological care. In complex or refractory cases, this distinction can help avoid empiric trials by guiding targeted treatment toward dominant mechanisms [38].

Higher diagnostic yield is clinically relevant only if it changes management. One study demonstrated that incorporating BSGM into routine specialist care aided management decisions in 84% of patients with chronic gastroduodenal symptoms, mainly through medication changes or diagnostic reclassification [39]. This value extends to medically complex patients. In a 10-patient case series of nutritionally compromised patients with suspected gut dysmotility, BSGM helped distinguish motor dysfunction from sensory or gut-brain mechanisms, updated the clinical diagnosis in 6/10, and guided PN weaning in 6/9 within a multidisciplinary care pathway [40]. Accordingly, observational studies also reported lower healthcare use after BSGM, including a 50% reduction in mean annual costs and more than US$7,000 in annualized savings per patient [39, 41] These findings suggest that better phenotyping may reduce repeated low-yield testing and empiric treatment cycling.

Although emerging, BSGM may also help identify patients likely to respond to specific therapies. In one cohort study, among 42 patients treated with prokinetics, low postprandial amplitude predicted a symptomatic response, whereas those with gastric dysrhythmia were less likely to respond [42]. These findings suggest that neuromuscular phenotyping may help predict treatment response patterns. Preliminary G-POEM data have also shown a phenotype-specific response: dysrhythmic and continuous phenotypes were associated with response, whereas high gastric frequency predicted non-response [43]. While such findings are encouraging, further data from larger prospective multicentre trials are currently awaited to confirm and expand understanding.

Among the best data to date, however, are real-world evidence from a tertiary motility practice indicating that BSGM may have prognostic value. In a 153-patient cohort, abnormal BSGM motor phenotypes were associated with medical therapy failure, defined as the need for advanced intervention or nutritional support. This association was independent of delayed gastric emptying (OR 2.87, 95% CI 1.2–7.0) [44] [conference abstract, DDW 2026]. Failure was highest in patients with both delayed emptying and the dysrhythmia phenotype, occurring in 86.3% of such patients. This combined phenotype identifies patients who need earlier multidisciplinary care, nutritional planning, pyloric evaluation, or advanced intervention.

#### Pediatric and Adolescent Populations

Pediatric and adolescent data support a similar need for BSGM-based phenotyping [45], and a separate normative reference interval has now become available for patients aged 12–17 years [46]. Because BSGM is radiation-free and non-invasive, it may be particularly useful in children when conventional testing is less desirable.

In studies of patients aged 12–21 years, functional dyspepsia and gastroparesis showed overlapping symptoms and could not be distinguished by standard clinical measures, whereas BSGM identified three electrophysiologic phenotypes (normal, delayed meal response, and dysrhythmic/low amplitude), with differences in nausea, anxiety, and physical health [31]. Consistent with the real-world study discussed above, the dysrhythmic group represented a more severe cohort, with higher symptom burdens, disability scores and medical requirements. In a separate pediatric pilot, neuropathy defined by antroduodenal manometry (ADM), was concordant with BSGM dysrhythmia, suggesting that BSGM may serve as a screening test that could reduce reliance on invasive, time-consuming ADM in selected patients [32].

#### Additional Populations

BSGM phenotyping has also been studied in patients with diabetes, postsurgical conditions, and autonomic disorders, demonstrating broad emerging applications in elucidating symptom mechanisms. In long-standing type 1 diabetes, which may impact both ICC and induce neuropathy, symptomatic patients have less stable gastric rhythm than controls, with high-frequency phenotypes associated with the greatest upper GI symptom burden [33]. After sleeve gastrectomy, reduced frequency and amplitude are consistent with loss of the greater-curvature pacemaker region and may help explain symptoms when no mechanical obstruction is found [47]. After fundoplication, BSGM may help determine whether persistent symptoms reflect myoelectrical dysfunction or gut-brain symptom patterns, leading to different treatment choices [34]. In complex patients with suspected autonomic neuropathy, autonomic testing has been shown to complement BSGM and GES, particularly when symptoms are refractory or discordant with single-test results [48].

## Conclusions and Future Directions

Functional dyspepsia and gastroparesis describe clinical presentations, but effective treatment requires understanding the mechanisms underlying symptoms in individual patients. BSGM provides a non-invasive, multimodal assessment of gastric function that extends beyond gastric emptying studies and identifies reproducible physiologic phenotypes encompassing a spectrum from neuromuscular dysfunction to disorders of gut–brain interaction. By characterising gastric dysfunction more directly, BSGM offers information that may help explain symptom heterogeneity and guide further evaluation and treatment.

BSGM is nonetheless a relatively new addition to the diagnostic landscape, and several priorities remain for clinical translation. Foremost among these is more rigorously linking phenotypes to treatment response: ongoing studies are evaluating whether baseline BSGM metrics predict response to specific medications and whether BSGM improves patient selection for pyloric intervention. Further validation of the Auckland Classification v1.0 across larger multicentre cohorts will be needed to establish reproducibility and generalisability, with a refined v2.0 framework already anticipated in coming years. Mechanistic validation is also needed to determine whether specific phenotypes correspond to defined underlying abnormalities; for example, impaired accommodation in the low meal response phenotype, pyloric dysfunction in the emerging high-amplitude phenotype, and small-bowel pathology in the delayed onset symptoms phenotype. Longitudinal studies will also be important to determine whether phenotypes shift with disease progression and treatment.

Importantly, no single test will provide a complete mechanistic profile for every patient. The future of gastroduodenal diagnostics lies in the combined use of multiple complementary investigations, including BSGM, gastric emptying studies, and potentially additional modalities such as autonomic evaluations, EndoFLIP, and measures of accommodation, to build fully integrated patient phenotypes. As this multimodal approach matures, new nomenclature schemes and integrated phenotyping systems that synthesise data across tests are likely to be required. With over 50 BSGM studies published in the last few years and a rapidly expanding evidence base, this field promises a next generation of diagnostics for gastric motility disorders, moving clinical practice from empirical management toward mechanism-based, phenotype-guided care.

## Key References

- Pasricha PJ, Grover M, Yates KP, Abell TL, Bernard CE, Koch KL, et al. Functional Dyspepsia and Gastroparesis in Tertiary Care are Interchangeable Syndromes With Common Clinical and Pathologic Features. Gastroenterology. 2021;160(6):2006–17. 10.1053/j.gastro.2021.01.230.
  - Multicenter study demonstrating that functional dyspepsia and gastroparesis are interchangeable syndromes with shared pathology and poor diagnostic stability on repeat gastric emptying testing. Foundational to the argument that emptying-based classification is insufficient.
- Varghese C, Dachs N, Schamberg G, Abell TL et al. Expert clinical consensus on body surface gastric mapping phenotypes for gastroduodenal disorders: ‘Auckland Classification’ v1.0. Neurogastroenterology & Motility. 2026; Online ahead of press.
  - International consensus establishing the Auckland Classification v1.0, the first formal phenotype classification for Body Surface Gastric Mapping.
- Gharibans AA, Calder S, Varghese C, Waite S, Schamberg G, Daker C, et al. Gastric dysfunction in patients with chronic nausea and vomiting syndromes defined by a noninvasive gastric mapping device. Sci Transl Med. 2022;14(663):eabq3544. 10.1126/scitranslmed.abq3544.
  - Landmark study validating the BSGM device in patients with chronic nausea and vomiting syndromes, demonstrating that high-resolution body surface gastric mapping identifies gastric myoelectrical abnormalities and distinguishes clinical phenotypes not captured by conventional testing.
- Wang WJ, Foong D, Calder S, Schamberg G, Varghese C, Tack J, et al. Gastric Alimetry Expands Patient Phenotyping in Gastroduodenal Disorders Compared with Gastric Emptying Scintigraphy. Am J Gastroenterol. 2024;119(2):331–41. 10.14309/ajg.0000000000002528.
  - Head-to-head comparison of BSGM and gastric emptying scintigraphy showing significantly higher diagnostic yield with BSGM and complementary value of combined testing.

## Data Availability

No datasets were generated or analysed during the current study.
